# Results and Guidelines From a Repeated-Measures Design Experiment Comparing Standing and Seated Full-Body Gesture-Based Immersive Virtual Reality Exergames: Within-Subjects Evaluation

**DOI:** 10.2196/17972

**Published:** 2020-07-27

**Authors:** Wenge Xu, Hai-Ning Liang, Qiuyu He, Xiang Li, Kangyou Yu, Yuzheng Chen

**Affiliations:** 1 Xi'an Jiaotong-Liverpool University Suzhou China

**Keywords:** exergames, immersive virtual reality, standing exergame, seated exergame, exercising

## Abstract

**Background:**

Although full-body seated exercises have been studied in a wide range of settings (ie, homes, hospitals, and daycare centers), they have rarely been converted to seated exergames. In addition, there is an increasing number of studies on immersive virtual reality (iVR) full-body gesture-based standing exergames, but the suitability and usefulness of seated exergames remain largely unexplored.

**Objective:**

This study aimed to evaluate the difference between playing a full-body gesture-based iVR standing exergame and seated exergame in terms of gameplay performance, intrinsic motivation, and motion sickness.

**Methods:**

A total of 52 participants completed the experiment. The order of the game mode (standing and sitting) was counterbalanced. Gameplay performance was evaluated by action or gesture completion time and the number of missed gestures. Exertion was measured by the average heart rate (HR) percentage (AvgHR%), increased HR%, calories burned, and the Borg 6-20 questionnaire. Intrinsic motivation was assessed with the Intrinsic Motivation Inventory (IMI), whereas motion sickness was assessed via the Motion Sickness Assessment Questionnaire (MSAQ). In addition, we measured the fear of falling using a 10-point Likert scale questionnaire.

**Results:**

Players missed more gestures in the seated exergame than in the standing exergame, but the overall miss rate was low (2.3/120, 1.9%). The analysis yielded significantly higher AvgHR%, increased HR%, calories burned, and Borg 6-20 rating of perceived exertion values for the seated exergame (all *P*<.001). The seated exergame was rated significantly higher on peripheral sickness (*P*=.02) and sopite-related sickness (MSAQ) (*P*=.004) than the standing exergame. The score of the subscale “value/usefulness” from IMI was reported to be higher for the seated exergame than the standing exergame. There was no significant difference between the seated exergame and standing exergame in terms of intrinsic motivation (interest/enjoyment, *P*=.96; perceived competence, *P*=.26; pressure/tension, *P*=.42) and the fear of falling (*P*=.25).

**Conclusions:**

Seated iVR full-body gesture-based exergames can be valuable complements to standing exergames. Seated exergames have the potential to lead to higher exertion, provide higher value to players, and be more applicable in small spaces compared with standing exergames. However, gestures for seated exergames need to be designed carefully to minimize motion sickness, and more time should be given to users to perform gestures in seated exergames compared with standing exergames.

## Introduction

### Background

Physical inactivity has been identified as the fourth leading cause of death globally [1]. It is now well established that a sedentary lifestyle is a unique risk factor for several illnesses, such as type 2 diabetes and cardiovascular diseases, which account for about 30% of global mortality [2]. Exergames represent a promising approach that has been widely examined for various population groups (ie, children [3], young individuals [4], and older adults [5]) to promote regular exercise (defined as planned, structured, repetitive, and intentional movement intended to improve or maintain physical fitness) in unmotivated or inactive target groups [6,7].

In recent years, exergames have been proven to have the potential to improve enjoyment, motivation, and long-term engagement when compared with other conventional exercises (eg, cardiovascular exercises like biking [8,9]), and as such, they can be effective in promoting both physical and mental health [10,11]. Various nonimmersive virtual reality (VR) [12] (like using interfaces such as a flat-screen television/monitor) exergames have been designed to encourage people to be more active [5] and promote a positive lifestyle [13] and self-care [14]. Previous literature has shown that exergames could bring physical and health outcomes to players. For example, Peng et al [15] performed a meta-analysis of energy expenditure in exergames, and their main finding suggests that exergames are as effective as traditional exercises in facilitating light- and moderate-intensity physical exertion. Huang et al [16] reported that participants who were enthusiastic about exercising showed positive changes in happiness, perceived energy levels, and relaxation in a 2-week exergame intervention. Sapi et al [17] reported that participants showed improvements in balance following a 6-week exergame intervention, and the improvements were in favor of using the exergame than conventional balance training. da Silva Alves et al [18] found that participants showed improvements in functional well-being and physical well-being after 10 sessions of exergaming. In the study by Garcia et al [19], participants showed improvements in stepping, standing balance, gait speed, and mobility following a 12-week exergame intervention.

With the recent advances in immersive virtual reality (iVR) head-mounted displays (HMDs), an increasing number of iVR exergames [20-22] are being developed. They have opened the possibility of altering more radically how we engage users in performing exercises. Studies have shown that iVR has the potential to produce benefits that other types of displays (ie, a standard display like television) cannot offer. For instance, iVR exergames can offer users the illusion of more exceptional physical capabilities than they have. As such, iVR may increase motivation for exercising in general [23]. Moreover, iVR games can offer benefits such as increased perceived competence and the feeling of body movements that are more in line with how we perform exercise in the real world. Participants have described exaggerated movements to be natural, fun, and empowering [24]. Furthermore, exercising in iVR has been found to be an effective intervention to increase enjoyment and motivation than standard televisions or monitors [8,25], where enjoyment and motivation are, in turn, linked to increased adherence to physical exercises in general [26-28].

Full-body gesture-based exergames have been widely explored with people in standing positions in iVR [20,22,23]. However, they have not been adapted and explored in seated versions. Seated iVR exergames could have the following benefits: (1) suitability for users with a sedentary lifestyle (eg, university students [29]); (2) feasibility for mobility-impaired users (eg, elderly users and wheelchair users [5,30]); (3) possibility of reducing the risk of injuries due to falls or motion sickness [31,32]; and (4) avoidance of injury from hitting other objects (eg, furniture) when the space is small or surroundings are cluttered, because players are not required to walk around.

Motivation can be divided into intrinsic (enjoyment of the activity) and extrinsic (driven by external outcomes, eg, losing weight and improving fitness) [33]. Intrinsic motivation (ie, motivation derived from enjoyment and satisfaction gained from an activity) plays an essential role in long-term adherence to exercising [26,27,33], whereas extrinsic motivation, such as competitive pressure, may lead to tension and feelings of compulsion, and can diminish intrinsic motivation [34,35]. There is evidence that exergames increase enjoyment and intrinsic motivation compared with conventional exercises (eg, biking) and distract from uncomfortable bodily sensations [25,36-39].

A sedentary lifestyle is a problem for older adults and people with physical disabilities and is a serious health problem among university students [40]. Most of the research on exergames has been targeted at older adults or disabled people [5,30,41] but not university students, who are underrepresented in such studies. Research has shown that lack of time and not liking exercising are the major barriers for university students [29]. These barriers could be overcome by using full-body gesture-based exergames that can be played either standing or seated at any time and in small spaces because exergames are perceived to be more enjoyable and preferred by university students than other conventional exercises (eg, cardiovascular exercises like biking [8,9]).

### Goal of the Study

The focus of this research was to evaluate the playability and user experience of a seated iVR exergame compared with a similar standing exergame among university students in terms of gameplay performance (ie, action completion time and number of gestures missed) and user experience (ie, motion sickness, intrinsic motivation, and fear of falling).

## Methods

### Experiment Design

We employed a one-way within-subjects experiment design where the independent variable was *game mode* with two levels (standing and sitting). The order of the game mode was counterbalanced to compensate for any learning effects. The whole experiment lasted between 30 and 40 minutes for each participant depending on their tiredness level and resting heart rate (RestHR).

### Participants

Participants were recruited from a local university campus through posters, social media platforms, and a mailing list. The study included university students with the ability to speak English, who were not disabled, were not pregnant (because of the physical exertion required to play the game), and had not consumed any alcohol during the day (blood alcohol level of approximately 0.07% could reduce symptoms of cybersickness [42], which might affect the results of our study).

Participants were excluded from the experiment if they (1) answered “yes” to any of the Physical Activity Readiness Questionnaire (PAR-Q) [43] questions, (2) had a resting blood pressure higher than 140/90 mmHg, and (3) had a RestHR level that was too low (ie, RestHR <62 beats/min for a 16 to 19-year-old female, RestHR <60 beats/min for a 20 to 39-year-old female, RestHR <56 beats/min for a 16 to 19-year-old male, or RestHR <55 beats/min for a 20 to 39-year-old male) or too high (ie, RestHR >94 beats/min for a 16 to 19-year-old female, RestHR >89 beats/min for a 20 to 39-year-old female, RestHR >87 beats/min for a 16 to 19-year-old male, or RestHR >84 beats/min for a 20 to 39-year-old male) [44].

All participants received drinks and snacks for their participation after they finished the experiment. The University Ethics Committee at Xi’an Jiaotong-Liverpool University approved the experiment. All participants signed informed consent forms prior to taking part in the study, and the research protocol was approved by the University Ethics Committee.

To determine the sample size for the study, a statistical power analysis was performed. This statistical power analysis was not based on data from prior studies owing to limited similar work. It was based on general considerations about the trade-offs between the ability to detect certain effects and the feasibility to acquire a sufficiently large sample. We used a sample size calculation software program (G*Power version 3.1.9.2 for Windows), with an effect size of 0.5 (Cohen *d*), statistical power of .90, and statistical level of significance of .05. The sample size was established at 44 participants, and we decided to recruit an additional 10 participants in the case of dropouts.

### Game

The game was implemented in Unity3D with the SteamVR plugin to enable positional tracking of the HTC VIVE trackers and controllers.

#### Rules and Logic

A game is an activity that requires at least one player, has rules, and has a victory condition [45]. The design of our game followed this definition and was inspired by the game called *Just Dance*, which requires users to follow and imitate the dancing gestures one by one and has been used in some prior studies [46]. However, the level of our program consists of a sequence of exercise gestures instead of dance movements. As the game starts, the player needs to follow the body gestures of the instructor in the VR system to move his/her body accordingly. For ease of reproducing the gestures, a gesture is deemed completed if specific joint positions (eg, head, controllers, and trackers) of the player meet the predefined variables of corresponding gestures based on a simple rule-based system and if the player can keep the pose for 0.4 seconds, which was determined from the results of a pilot study with 10 participants, where we found that a short pose hold time could lead to gesture misrecognitions and a long pose hold time could lead to player fatigue easily. In addition, this time of 0.4 seconds was informed from the literature in other fields (eg, text entry [47]). A badge [48] is given to players when they complete every 10 actions as an in-game achievement to motivate them to follow and replicate the gestures carefully. The victory condition was to successfully follow the instructor’s gestures and not fail to follow these gestures three times in a row. In addition, our game warned users when they were not paying attention to the virtual instructor by tracking the rotation data from the HMD. Both visual and auditory feedback were provided to encourage players to continue playing.

#### Game Procedure

**Figure 1:**
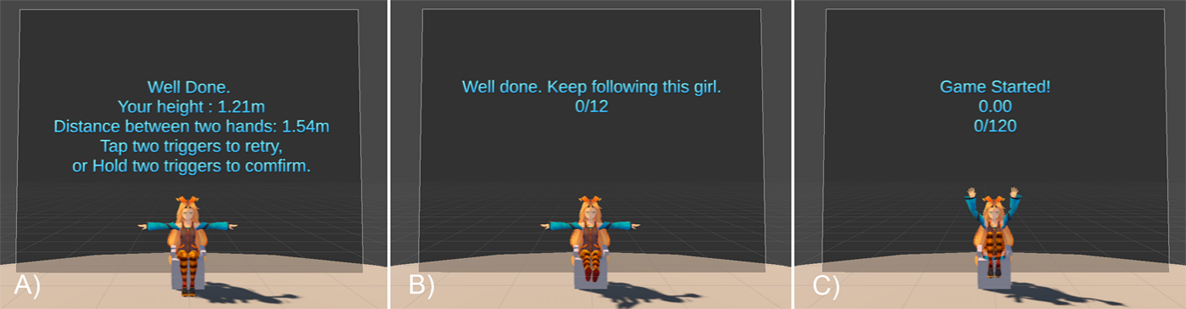
(A) Calibration, (B) training, and (C) game phases for the seated version; the process for the standing version is the same, except the instructor is standing instead of sitting.

The game starts with a calibration phase (Figure 1A) for the system to take into account the individual differences of players. The player needs to lift the hands midair and confirm having finished the calibration by pressing a button on the controllers. The system then records the position data of the head, hands, and feet. After the calibration phase, the game progresses to the training (warm-up) phase (Figure 1B), where the player needs to follow the virtual instructor to perform two rounds of six gestures with a fixed order to become familiar with the gestures that need to be performed. The gameplay phase (Figure 1C) starts after the training phase, where the player needs to follow the virtual instructor who performs gestures presented in a random manner.

#### Included Gestures

**Figure 2:**
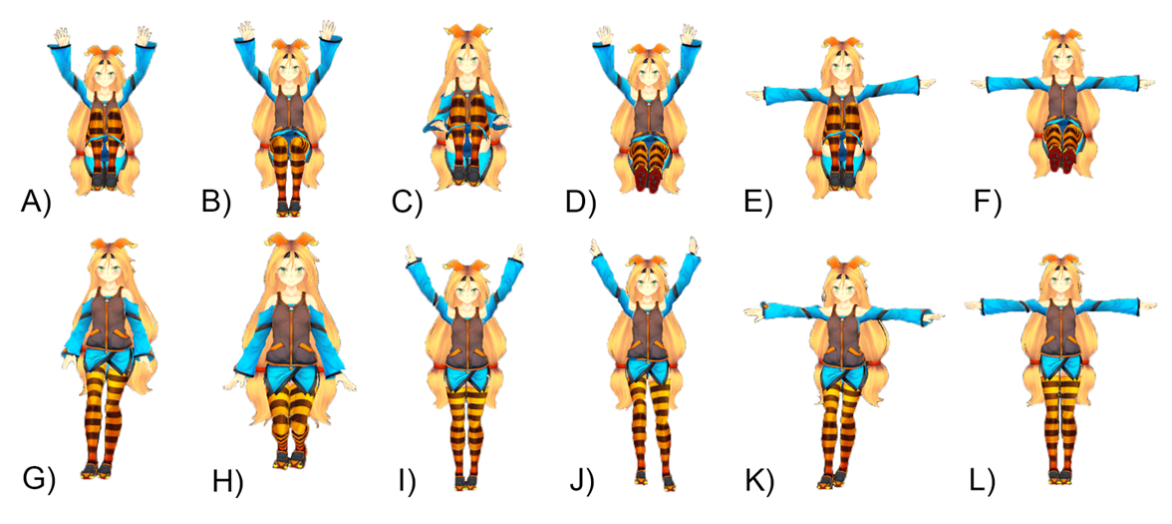
Seated gestures: (A) Hands up + knees up; (B) Hands up; (C) Knees up; (D) Feet up + hands up; (E) Hands stretched + knees up; (F) Hands stretched + feet up. Standing gestures: (G) Left/right kick; (H) Squat; (I) Hands up; (J) Left/right kick + hands up; (K) Left/right kick + hands stretched; (L) Hands stretched.

Table 1 shows the intensity of the gestures selected for our game. Figure 2 shows the final pose of the gestures involved in the sitting and standing versions of the exergame. The selection process of gestures and the intensity values were informed by the results of a pilot study (Multimedia Appendix 1).

**Table 1:** Intensity level of the gestures used in the experiment.

Gesture		Intensity (%)
**Sitting gesture**		
	Hands up	32.30
	Knees up	32.27
	Feet up + hands up	35.10
	Feet up + hands stretched	34.75
	Knees up + hands up	42.39
	Knees up + hands stretched	44.03
**Standing gesture**		
	Hands up	31.00
	Hands stretched	37.20
	Left/right kick	27.25
	Squat	50.69
	Hand stretched + kick	43.88
	Hands up + kick	46.05

### Outcome Measurements

#### Exertion

We measured participants’ exertion based on HR and calories burned using a Polar OH1 wrist-strap monitor. Average HR (AvgHR%) was expressed as a percentage of a participant’s estimated maximum HR (MaxHR), where MaxHR was estimated as 220 minus age [49]. This measure is commonly used in exercise studies to confirm that participants are working at a required level of exertion. Additionally, we measured the increased HR%, which was the difference between the HR% at the beginning and the end of the game phase, for a direct comparison of both versions. Calories burned were calculated using the Polar Beat mobile app with the activity set as other indoor activity and the user profile of the app calibrated to each participant. We started recording the HR and calories burned as soon as the participants finished the training phase. Furthermore, the Borg CR 6-20 [50] rating of perceived exertion (RPE) was used to measure the participants’ perceived exertion level. It describes the physical efforts involved in completing the game as perceived by the participants, with “6” indicating “no exertion,” “13” indicating “somewhat hard exertion,” and “20” indicating “maximum exertion.” Borg RPE is frequently used in exercise sciences as a quantitative measure of perceived exertion when exercising [50,51], and it has been applied to studies with iVR exergames [4,20].

#### Gameplay Data

We collected several types of data in the background, including (1) the action completion time of every successfully performed gesture, which was equal to the time spent by the user to perform the same pose and hold the pose for 0.4 seconds, (2) the number of gestures missed for each gesture type, and (3) the real-time HR data from the Polar OH1 optical HR sensor for every 0.2 seconds in the actual experiment stage. Therefore, we analyzed (1) the average action completion time, (2) the total number of missed gestures, and (3) the plot profile of real-time HR.

#### Motion Sickness

Motion sickness was assessed using the self-reported 16-item Motion Sickness Assessment Questionnaire (MSAQ) [52], which is a valid descriptor of motion sickness in the general population that covers the following four dimensions of motion sickness: gastrointestinal (stomach sick, queasy, nauseated, and vomit), central (faint-like, lightheaded, dizzy, spinning, and disoriented), peripheral (sweaty, clammy, and hot/warm), and sopite-related (annoyed, drowsy, tired, and uneasy). The results from the MSAQ were correlated strongly with the overall scores from the Pensacola diagnostic index (*r*=0.81; *P*<.001) and the nausea profile (*r*=0.92; *P*<.001) [52]. It has been found that the MSAQ is a valid evaluation tool and that it is advantageous to use this multidimensional questionnaire rather than the one-dimensional form [52]. The questionnaire has been widely used in studies dealing with virtual environments [53-55]. The scale ranges from 1 (not at all) to 9 (severely). A lower score is associated with lower motion sickness.

#### Intrinsic Motivation

Intrinsic motivation was measured using the self-reported 25-item version of the Intrinsic Motivation Inventory (IMI) [56], which covers the following four subscales: interest/enjoyment, perceived competence, pressure/tension, and value/usefulness. Although IMI includes seven subscales, only interest/enjoyment measures intrinsic motivation and is considered the primary self-reporting measure. We included the perceived competence and pressure/tension subscales because they are positive and negative predictors of intrinsic motivation, respectively. In addition, the subscale value/usefulness has been used in internalization studies [57] and can provide us with an idea about how people internalize and self-regulate themselves with respect to the activities that they experience as useful or valuable for themselves. The IMI has gained widespread acceptance as a multidimensional measure of intrinsic motivation in the context of sports and exercising [58,59] and has been widely used in studies dealing with iVR exergames [23,60,61]. Each item was rated on a severity scale ranging from 1 (not at all) to 7 (very). A higher score indicates a more internally motivated self-regulated physical activity behavior.

We measured fear of falling by asking participants “how concerned are you about the possibility of falling during the experiment?” using a 10-point Likert scale from 1 to 10, with 1 indicating “very slightly or not at all” and 10 indicating “extremely.”

After completing the above questionnaires, participants were asked to answer the following open-ended question in the questionnaire: “What do you think about this version of the game?” They responded by typing into a text box. There was no limit for the length of participants’ responses. A full list of questions used after each condition can be found in Multimedia Appendix 2.

### Apparatus and Setup

**Figure 3:**
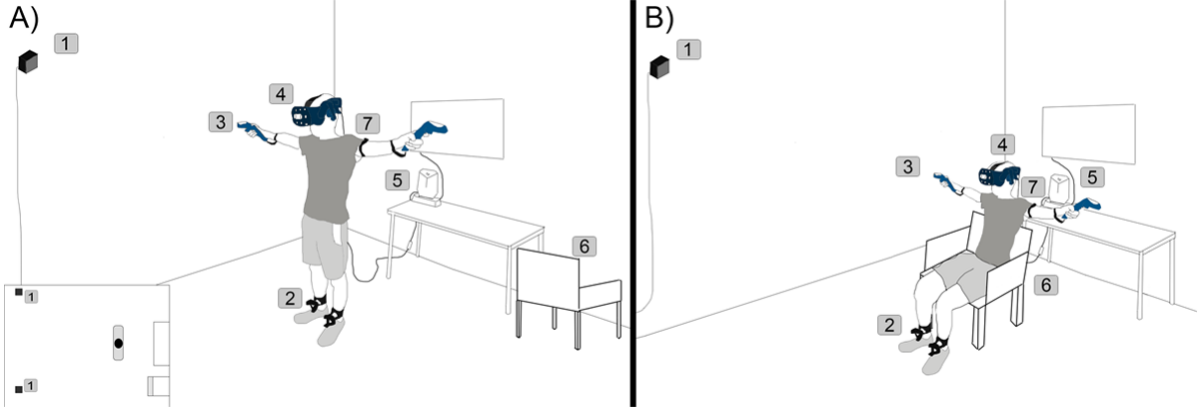
Standing (A) and seated (B) experiment setup, and the devices used in the experiment: (1) the HTC VIVE base stations, the locations of the two base stations can be found in the vertical view in the left bottom corner; (2) the two VIVE trackers attached to the legs; (3) the VIVE controllers used to track the player’s hand positions; (4) HTC VIVE Pro Eye; (5) the HP Z backpack; (6) the chair; and (7) Polar OH1.

The experiment was conducted using HTC VIVE Pro Eye connected to an HP Z workstation (i7 CPU, 16 GB RAM, and Nvidia Quadro P5200 GPU). Two HTC VIVE handheld controllers, two HTC VIVE trackers, and two base stations were used to enable hand and feet motion tracking. A stable chair with two handles was used in the sitting condition. The HR was monitored by a Polar OH1 optical HR sensor, which has been proved to be able to capture good HR data when compared with the gold standard of HR measurement with an electrocardiography device [62,63]. The experiment was conducted in an indoor laboratory room that could not be seen from the outside. The room temperature was always set to be 23 to 24°C during the experiment. Figure 3 depicts the experiment setup and devices used in the experiment.

### Procedure

Before filling in the pre-experiment questionnaire that gathered demographic information (eg, age, gender, and experience with the VR device), we obtained participants’ consent to participate in the experiment and collected their RestHR and resting blood pressure. They were also asked to enter their age, gender, height, and weight into the Polar Beat app. Before each condition started, a researcher would help them to wear the VIVE Pro Eye headset with two VIVE handheld controllers and two VIVE trackers. Participants were then given 3 minutes to get familiar with the corresponding condition of the iVR exergame. Once their HR reached the equivalent RestHR level, they proceeded to the experiment stage, beginning with calibrating the game, training, and testing. In each condition, they needed to perform 120 gestures, requiring 5 minutes (120 gestures × 2.5 seconds). We fixed the number of gestures to allow for comparing the two games. After each condition, they were asked to fill in postexperiment questionnaires. They proceeded to the next condition when they felt rested and their HR was at the rest level.

### Statistical Analysis

We used the paired *t* test to understand the difference between the seated exergame and standing exergame regarding gameplay performance, IMI, MSAQ, fear of falling, and exertion measurements. For the percentage of missed gesture types for sitting/standing gesture type, we used one-way repeated analysis of variance (ANOVA) with the sitting/standing gesture type as the within-subjects variables, respectively. We also examined and reported if there were any significant gender differences in our measurements by using one-way between-subjects ANOVA. We set the α level at .05 in our analyses. We further reported the effect sizes using Cohen suggestion to classify the effect size, where Cohen suggested that *d*=0.2 represents a “small” effect size, 0.5 represents a “medium” effect size, and 0.8 represents a “large” effect size [64]. Analyses were performed using the Statistical Package for the Social Sciences (IBM Corp).

## Results

### Participant Characteristics

Fifty-four individuals were interested in participating in the experiment. Two were excluded owing to their high RestHR. At the end, a total of 52 participants were eligible to participate in the study. The characteristics of the study participants are shown in Table 2.

**Table 2:** Characteristics of the study participants.

Characteristic		Value
Number of students, n		52
Age (years), mean (SD)		18.81 (1.70)
RestHR^a^, mean (SD)		77.71 (8.78)
Height (cm), mean (SD)		170.11 (7.75)
Weight (kg), mean (SD)		60.84 (10.79)
Body mass index (kg/m^2^), mean (SD)		20.93 (2.87)
Self-reported exercise time per week (min), mean (SD)		87.88 (66.55)
Normal or corrected-to-normal, n		52
Self-reported experience with seated exercise regimes		No
**Self-reported experience with VR^b^ HMDs^c^, n**		21
	Frequent user, n	1
**Self-reported experience with full-body gesture-based video games, n**		28
	Frequent player, n	4

^a^RestHR: resting heart rate.

^b^VR: virtual reality.

^c^HMD: head-mounted display.

### Gameplay Data

Gameplay data and analysis results are reported in Table 3. The analysis showed that game mode did not influence action completion time. However, the analysis showed that players missed more gestures in the seated exergame than in the standing exergame.

**Table 3:** Gameplay data and exertion measures.

Variable		Standing exergame, mean (SD)	Seated exergame, mean (SD)	t_51_	*P* value^a^	Cohen *d*
**Gameplay**						
	Action completion time	1.48 (0.14)	1.53 (0.18)	1.87	.07	N/A^b^
	Missed gestures	1.65 (1.71)	2.33 (1.83)	2.40	.02	0.332
**Exertion**						
	AvgHR%^c^	51.9% (4.6%)	54.3% (5.0%)	4.66	<.001	0.646
	Increased HR%^d^	6.9% (4.4%)	11.8% (5.3%)	5.86	<.001	0.813
	Calories	21.83 (6.76)	24.67 (7.25)	4.44	<.001	0.615
	Borg 6-20	9.02 (2.15)	10.25 (2.59)	3.96	<.001	0.548

^a^Significant at .05.

^b^N/A: not applicable.

^c^AvgHR%: average heart rate percentage.

^d^HR%: heart rate percentage.

#### Percentage of Missed Gesture Types

**Figure 4:**
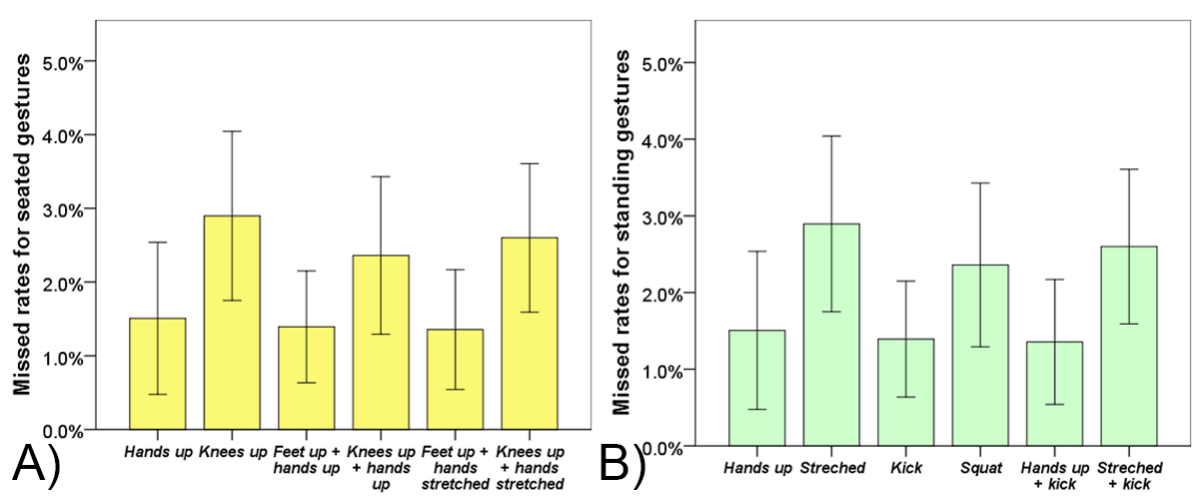
Missed rates for (A) seated gestures and (B) standing gestures. Error bars indicate ±2 standard errors.

The results of one-way repeated ANOVA yielded no significant effect of the sitting gesture type (*F*_5,255_=1.98, *P*=.08) or standing gesture type (*F*_5,255_=1.058, *P*=.38) on the percentage of corresponding missed gestures. The missed rate for sitting and standing gesture types can be found in Figure 4.

### Exertion

Exertion (AvgHR%, increased HR%, calories burned, and Borg 6-20) data and analysis results are presented in Table 3. The analysis yielded significantly higher AvgHR%, increased HR%, calories burned, and Borg 6-20 RPE for the seated exergame (all *P*<.001). Our results suggest that the seated exergame was rated as “very light” to “light” exercise and the standing exergame was rated as “very light” exercise according to the Borg 6-20 RPE scale.

**Figure 5:**
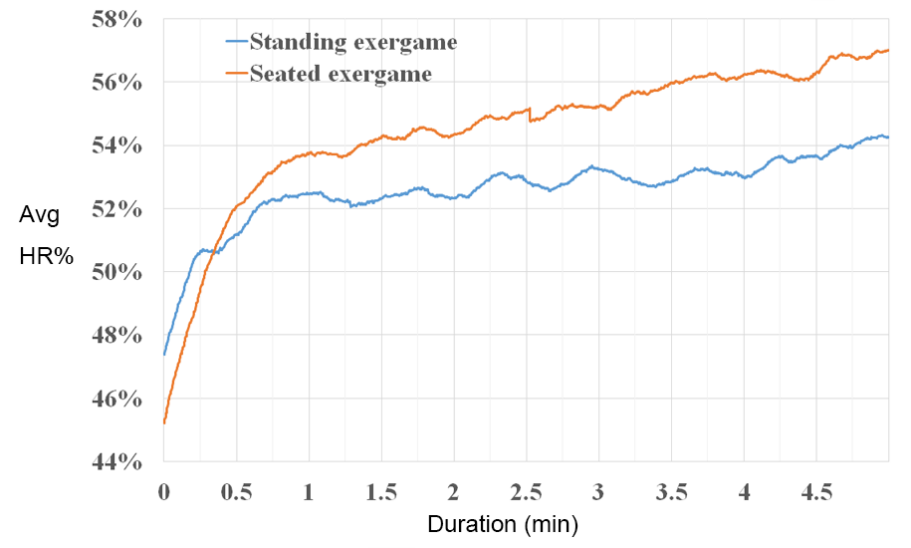
Mean AvgHR% during gameplay for both versions of the exergame; the interaction between two lines occurs at 0.38 minutes. At 2.64 minutes, AvgHR% of the seated exergame reached the moderate physical intensity level. AvgHR%: average heart rate percentage.

To aid the visualization of the AvgHR% behavior of both exergames, Figure 5 shows the AvgHR% data from all participants during the 5 minutes of gameplay, averaged over the whole session. The seated exergame had a higher AvgHR% than the standing exergame after 0.34 minutes.

### Experience

Data analysis of the MSAQ included the overall MSAQ score and its subscale scores (gastrointestinal, central, peripheral, and sopite-related). The MSAQ data and analysis results are reported in Table 4. The analysis showed significantly higher peripheral sickness (*P*=.02) and sopite-related sickness (*P*=.004) for the seated exergame. We did not find a significant difference between the seated exergame and standing exergame in terms of central (*P*=.81), gastrointestinal (*P*=.81), and overall sickness (*P*=.06).

Regarding IMI, there was no significant difference for interest/enjoyment (*P*=.96), perceived competence (*P*=.26), and pressure/tension (*P*=.42). However, the analysis yielded a significantly higher value/usefulness for the seated exergame (*P*=.04). IMI data and analysis results can be found in Table 4. The one-way between-subjects ANOVA showed that interest/enjoyment was rated significantly higher by females (mean 5.20, SD 1.23) than males (mean 4.38, SD 41.16), with a medium effect size (*F*_1,50_=6.01, *P*=.02).

**Table 4:** Motion Sickness Assessment Questionnaire and Intrinsic Motivation Inventory test measures.

Variable			Standing exergame, mean (SD)		Sitting exergame, mean (SD)		t_51_		*P* value^a^		Cohen *d*
**MSAQ^b^**											
	Peripheral	22.7% (12.9%)		27.7% (14.3%)		−2.41		.02^a^		0.334	
	Sopite-related	18.8% (11.3%)		23.9% (15.4%)		−3.06		.004^a^		0.424	
	Central	19.2% (11.1%)		18.8% (10.7%)		0.24		.81		N/A^c^	
	Gastrointestinal	13.5% (5.7%)		13.7% (5.2%)		−0.24		.81		N/A	
	Overall	17.4% (7.3%)		19.4% (8.4%)		−1.91		.061		N/A	
**IMI^d^**											
	Interest/enjoyment	4.73 (1.30)		4.72 (1.30)		0.05		.96		N/A	
	Competence	4.99 (1.12)		4.85 (1.20)		1.14		.26		N/A	
	Pressure/tension	2.90 (0.95)		2.99 (1.04)		−0.82		.42		N/A	
	Value/usefulness	5.12 (1.28)		5.38 (1.12)		−2.11		.04^a^		0.292	

^a^Significant at .05.

^b^MSAQ: Motion Sickness Assessment Questionnaire.

^c^N/A: not applicable.

^d^IMI: Intrinsic Motivation Inventory.

#### Fear of Falling

There was no significant difference in the fear of falling ratings between the standing exergame (mean 2.10, SD 1.58) and seated exergame (mean 2.40, SD 1.78) (t_51_=−1.16, *P*=.25).

## Discussion

### Overview

With the limited exploration of seated exergames in the literature of iVR exergames, this study is the first to explore the difference between full-body gesture-based seated exergames and standing exergames in iVR among university students regarding playability (ie, gameplay performance) and user experience (ie, intrinsic motivation and motion sickness). Our results suggest that participants perceived higher value in the seated exergame than in the standing exergame. However, the seated exergame was associated with a worse gameplay performance (ie, the number of missed gestures) and a higher rating of motion sickness than the standing exergame.

Although we observed that participants missed a significantly higher number of gestures in the seated exergame than in the standing exergame (*P*=.02), this rate was as low as 1.9% (2.3/120). Further analysis of the type of gestures missed in the seated exergame confirmed that these misses were in the early stages of the experiment, and as such, the main reason for the misses could be because of participant unfamiliarity with exercising in the seated position (none of them had previous experience of seated exercising; Table 2).

Regarding motion sickness, previous studies [31,32] have suggested that the seated exergame might result in a lower level of motion sickness. However, this was not supported by our findings. We observed that participants felt sicker (ie, peripheral and sopite-related motion sickness) in the seated exergame. However, the reason was beyond the scope of this study; a further investigation is required to understand why motion sickness was higher in the seated exergame than in the standing exergame. We suggest that future designers and researchers should carefully design full-body gestures for iVR seated exergames to minimize motion sickness.

As for intrinsic motivation, we did not observe any significant difference between the seated exergame and standing exergame (ie, interest/enjoyment, *P*=.96; perceived competence, *P*=.26; pressure/tension, *P*=.42). However, there was a gender effect on participants’ intrinsic motivation toward exergames, where we found that females had a significantly higher intrinsic motivation (ie, interest/enjoyment) than males (*P*=.02). This could be because the exergame involved in our study was more like a dance game, which was inspired by *Just Dance*, and prior research [65] has shown that females tend to be more physically active in dance exergames. Aside from this difference between male and female participants, no other differences were found in our experiment.

In most cases, standing exercises have a higher exercise intensity (in traditional exercises [66,67] and exergames [68,69]). We found that our seated exergame led to a higher exertion (ie, AvgHR%, increased HR%, calories burned, and Borg 6-20) than the standing exergame, possibly because the seated exergame involved more whole-body movements that required increased energy expenditure during gameplay [70-73].

### Design Guidelines

In this section, we provide design guidelines that are based on suggestions provided by Wiemeyer et al [74] for future game designers who are interested in building iVR full-body standing or seated gesture-based exergames.

First, practice should be provided for each gesture during warm-up. A warm-up session before exercising is essential [75], and it should be included in exergames as well. One way to perform warm-up for full-body gesture-based exergames is to practice the gestures involved in the game, which will not only help players reduce the risk of injures but also make them familiar with the in-game gestures.

Second, the difficulty level of the game should be adapted to the current state of the individual. Regarding an *offline* approach, players might have difficulty in performing certain gestures during gameplay. Therefore, to match the difficulty of the game to the current state of the individual, it would be necessary for players to experience and select gestures they are comfortable performing before playing the game. Regarding an *online* approach, one of the adaptive methods that has been used and proven to be suitable in exergames is proportional–integral–derivative (PID) control [76]. Designers can use PID control to modify the transition time between gestures or select the gestures based on the player’s real-time HR and gameplay performance (ie, the number of gestures missed). PID control is also useful to avoid overly vigorous exercise, which might put the exerciser at risk of eliciting unwanted coronary issues [77].

Third, warning signs should be provided for standing exergames if users have left (or are about to leave) the calibration position and are too far to keep them protected. This is because players tend to move around during gameplay, which we encountered in our study and has been reported previously [23]. It could lead to potentially dangerous situations (eg, hitting objects that are in the environment and going out of the safe tracking area) or decrease the recognition performance of the sensors (eg, tracking may not work when they are too close to or far from the sensors).

### Limitations and Future Work

Our study only focused on one sedentary lifestyle user group (university students). Future work could focus on investigating the two versions of the exergame with different population groups (eg, older adults and users who have physical disabilities). To minimize players’ cognitive workload, we set both exergames to include only 6 out of the 10 gesture types that we measured during the pilot study. In the future, we could add more gestures to increase the complexity of the game (as stated by participants 13, 20, 30, and 37). A further limitation is that our experiment did not measure which types of gestures caused the unwanted level of motion sickness in the seated exergame. Future experiments could be conducted to check issues related to motion sickness based on specific gestures and types of gestures.

### Conclusions

Our contributions to the field of iVR exergaming regarding gameplay performance and user experience are as follows: (1) the iVR seated exergame could result in higher exertion and provide higher value to players than the standing exergame; (2) participants might feel sicker in the iVR seated exergame than in the standing exergame, and as such, full-body gestures for seated exergames need to be designed carefully to help minimize the feeling of motion sickness; and (3) participants might miss more gestures in the iVR seated exergame than in the standing exergame. Therefore, designers should allow more time for performing gestures in the seated exergame.

## Appendix

Multimedia Appendix 1 Approach for measuring the intensity of gestures.

Multimedia Appendix 2 Questionnaire used in the study.

## Abbreviations

ANOVA: analysis of variance
AvgHR%: average heart rate percentage
HMD: head-mounted display
HR: heart rate
IMI: Intrinsic Motivation Inventory
iVR: immersive virtual reality
MaxHR: maximum heart rate
MSAQ: Motion Sickness Assessment Questionnaire
PAR-Q: Physical Activity Readiness Questionnaire
PID: proportional–integral–derivative
RestHR: resting heart rate
RPE: rating of perceived exertion
VR: virtual reality

## References

[1] Lee I, Shiroma EJ, Lobelo F, Puska P, Blair SN, Katzmarzyk PT (2012). Effect of physical inactivity on major non-communicable diseases worldwide: an analysis of burden of disease and life expectancy. The Lancet.

[2] Williams G, Fruhbeck G (2009). Obesity: Science to Practice.

[3] Hernandez H, Ye Z, Graham T, Fehlings D, Switzer L (2013). Designing action-based exergames for children with cerebral palsy. Proceedings of the SIGCHI Conference on Human Factors in Computing Systems.

[4] Xu W, Liang H, Zhang Z, Baghaei N (2020). Studying the Effect of Display Type and Viewing Perspective on User Experience in Virtual Reality Exergames. Games Health J.

[5] Gerling K, Livingston I, Nacke L, Mandryk R (2012). Full-body motion-based game interaction for older adults. Proceedings of the SIGCHI Conference on Human Factors in Computing Systems.

[6] Bogost I (2005). The Rhetoric of Exergaming. Proceedings of the Digital Arts and Cultures.

[7] Sinclair J, Hingston P, Masek M (2007). Considerations for the design of exergames. Proceedings of the 5th International Conference on Computer Graphics and Interactive Techniques in Australia and Southeast Asia.

[8] Farrow M, Lutteroth C, Rouse PC, Bilzon JL (2019). Virtual-reality exergaming improves performance during high-intensity interval training. Eur J Sport Sci.

[9] Moholdt T, Weie S, Chorianopoulos K, Wang AI, Hagen K (2017). Exergaming can be an innovative way of enjoyable high-intensity interval training. BMJ Open Sport Exerc Med.

[10] Pluchino A, Lee SY, Asfour S, Roos BA, Signorile JF (2012). Pilot study comparing changes in postural control after training using a video game balance board program and 2 standard activity-based balance intervention programs. Arch Phys Med Rehabil.

[11] Rosenberg D, Depp CA, Vahia IV, Reichstadt J, Palmer BW, Kerr J, Norman G, Jeste DV (2010). Exergames for Subsyndromal Depression in Older Adults: A Pilot Study of a Novel Intervention. The American Journal of Geriatric Psychiatry.

[12] Zeng N, Pope Z, Lee J, Gao Z (2018). Virtual Reality Exercise for Anxiety and Depression: A Preliminary Review of Current Research in an Emerging Field. J Clin Med.

[13] Gao Y, Mandryk RL, Anacleto JC, Fels S, Graham N, Kapralos B, Saif El-Nasr M, Stanley K (2011). GrabApple: The Design of a Casual Exergame. Entertainment Computing – ICEC 2011.

[14] Faiola A, Kharrazi H (2010). Diabetes education and serious gaming: Teaching adolescents to cope with diabetes. Health Informatics: A Patient-Centered Approach to Diabetes.

[15] Peng W, Lin J, Crouse J (2011). Is playing exergames really exercising? A meta-analysis of energy expenditure in active video games. Cyberpsychol Behav Soc Netw.

[16] Huang H, Nguyen HV, Cheng T, Wong M, Chiu H, Yang Y, Teng C (2019). A Randomized Controlled Trial on the Role of Enthusiasm About Exergames: Players' Perceptions of Exercise. Games Health J.

[17] Sápi M, Domján A, Fehérné Kiss A, Pintér S (2019). Is Kinect Training Superior to Conventional Balance Training for Healthy Older Adults to Improve Postural Control?. Games Health J.

[18] da Silva Alves R, Iunes DH, de Carvalho JM, Menezes FD, Silva AM, Borges JB, Carvalho LC (2018). Effects of Exergaming on Quality of Life in Cancer Patients. Games for Health Journal.

[19] Garcia JA, Schoene D, Lord SR, Delbaere K, Valenzuela T, Navarro KF (2016). A Bespoke Kinect Stepping Exergame for Improving Physical and Cognitive Function in Older People: A Pilot Study. Games Health J.

[20] Xie B, Zhang Y, Huang H, Ogawa E, You T, Yu L (2018). Exercise Intensity-Driven Level Design. IEEE Trans. Visual. Comput. Graphics.

[21] Xu W, Liang H, Zhao Y, Yu D, Monteiro D (2019). DMove: Directional Motion-based Interaction for Augmented Reality Head-Mounted Displays. Proceedings of the SIGCHI Conference on Human Factors in Computing Systems.

[22] Xu W, Liang H, Yu Y, Monteiro D, Hasan K, Fleming C (2019). Assessing the Effects of a Full-body Motion-based Exergame in Virtual Reality. Proceedings of the Seventh International Symposium of Chinese CHI.

[23] Ioannou C, Archard P, O?Neill E, Lutteroth C (2019). Virtual Performance Augmentation in an Immersive Jump & Run Exergame. Proceedings of the SIGCHI Conference on Human Factors in Computing Systems.

[24] Granqvist A, Takala T, Takatalo J, Hämäläinen P (2018). Exaggeration of Avatar Flexibility in Virtual Reality. Proceedings of the Annual Symposium on Computer-Human Interaction in Play.

[25] Shaw LA, Marks S, Wunsche BC, Lutteroth C, Buckley J, Corballis P (2015). Development and Evaluation of an Exercycle Game Using Immersive Technologies. Proceedings of the 8th Australasian Workshop on Health Informatics and Knowledge Management.

[26] Alexandris K, Tsorbatzoudis C, Grouios G (2017). Perceived Constraints on Recreational Sport Participation: Investigating their Relationship with Intrinsic Motivation, Extrinsic Motivation and Amotivation. Journal of Leisure Research.

[27] Frederick C, Ryan R (1993). Differences in Motivation for Sport and Exercise and their Relations with Participation and Mental Health. Journal of Sport Behavior.

[28] Ryan R, Frederick C, Lepes D, Rubio N, Sheldon K (1997). Intrinsic Motivation and Exercise Adherence. International Journal of Sport Psychology.

[29] Gómez-López M, Gallegos AG, Extremera AB (2010). Perceived barriers by university students in the practice of physical activities. J Sports Sci Med.

[30] Gerling K, Hicks K, Kalyn M, Evans A, Linehan C (2016). Designing Movement-based Play With Young People Using Powered Wheelchairs. Proceedings of the SIGCHI Conference on Human Factors in Computing Systems.

[31] Bolton J, Lambert M, Lirette D, Unsworth B (2014). PaperDude: a virtual reality cycling exergame. Proceedings of the SIGCHI Conference on Human Factors in Computing Systems-Extended Abstracts.

[32] Shaw L, Wunsche B, Lutteroth C, Marks S, Callies R (2015). Challenges in Virtual Reality Exergame Design. Proceedings of the Australasian User Interface Conference.

[33] Ryan RM, Deci EL (2000). Intrinsic and Extrinsic Motivations: Classic Definitions and New Directions. Contemp Educ Psychol.

[34] Deci E, Ryan R (1985). Intrinsic Motivation and Self-Determination in Human Behavior.

[35] Reeve J, Deci EL (1996). Elements of the Competitive Situation that Affect Intrinsic Motivation. Pers Soc Psychol Bull.

[36] Finkelstein S, Suma EA (2011). Astrojumper: Motivating Exercise with an Immersive Virtual Reality Exergame. Presence: Teleoperators and Virtual Environments.

[37] Bailey BW, McInnis K (2011). Energy cost of exergaming: a comparison of the energy cost of 6 forms of exergaming. Arch Pediatr Adolesc Med.

[38] Baños RM, Escobar P, Cebolla A, Guixeres J, Alvarez Pitti J, Lisón JF, Botella C (2016). Using Virtual Reality to Distract Overweight Children from Bodily Sensations During Exercise. Cyberpsychol Behav Soc Netw.

[39] Monedero J, Lyons EJ, O'Gorman DJ (2015). Interactive video game cycling leads to higher energy expenditure and is more enjoyable than conventional exercise in adults. PLoS One.

[40] Irwin JD (2007). The prevalence of physical activity maintenance in a sample of university students: a longitudinal study. J Am Coll Health.

[41] Xu W, Liang H, Baghaei N, Wu Berberich B, Yue Y (2020). Health Benefits of Digital Videogames for the Aging Population: A Systematic Review. Games Health J.

[42] Iskenderova A, Weidner F, Broll W (2017). Drunk Virtual Reality Gamingxploring the Influence of Alcohol on Cybersickness. Proceedings of the Annual Symposium on Computer-Human Interaction in Play.

[43] Thomas S, Reading J, Shephard RJ (1992). Revision of the Physical Activity Readiness Questionnaire (PAR-Q). Can J Sport Sci.

[44] Ostchega Y, Porter KS, Hughes J, Dillon CF, Nwankwo T (2011). Resting pulse rate reference data for children, adolescents, and adults: United States, 1999-2008. Natl Health Stat Report.

[45] Rogers S (2014). Level Up! The Guide to Great Video Game Design.

[46] Lin J (2015). "Just Dance": The Effects of Exergame Feedback and Controller Use on Physical Activity and Psychological Outcomes. Games Health J.

[47] Xu W, Liang H, Zhao Y, Zhang T, Yu D, Monteiro D, Yue Y (2019). RingText: Dwell-free and hands-free Text Entry for Mobile Head-Mounted Displays using Head Motions. IEEE Trans Vis Comput Graph.

[48] Farzan R, DiMicco J, Millen D, Dugan C, Geyer W, Brownholtz E (2008). Results from Deploying a Participation Incentive Mechanism within the Enterprise. Proceedings of the SIGCHI Conference on Human Factors in Computing Systems.

[49] Kluwer W (2013). ACSM's Guidelines for Exercise Testing and Prescription.

[50] Borg GA (1982). Psychophysical bases of perceived exertion. Med Sci Sports Exerc.

[51] Borg G (1970). Perceived exertion as an indicator of somatic stress. Scand J Rehabil Med.

[52] Gianaros PJ, Muth ER, Mordkoff JT, Levine ME, Stern RM (2001). A questionnaire for the assessment of the multiple dimensions of motion sickness. Aviat Space Environ Med.

[53] Speicher M, Feit A, Ziegler P, Krüger A (2018). Selection-based Text Entry in Virtual Reality. Proceedings of the SIGCHI Conference on Human Factors in Computing Systems.

[54] Xu W, Liang H, He A, Wang Z (2019). Pointing and Selection Methods for Text Entry in Augmented Reality Head Mounted Displays. Proceeding of IEEE International Symposium on Mixed and Augmentation Reality.

[55] Frederiksen JG, Sørensen SM, Konge L, Svendsen MB, Nobel-Jørgensen M, Bjerrum F, Andersen SA (2020). Cognitive load and performance in immersive virtual reality versus conventional virtual reality simulation training of laparoscopic surgery: a randomized trial. Surg Endosc.

[56] Ryan RM (1982). Control and information in the intrapersonal sphere: An extension of cognitive evaluation theory. Journal of Personality and Social Psychology.

[57] Deci EL, Eghrari H, Patrick BC, Leone DR (1994). Facilitating internalization: the self-determination theory perspective. J Pers.

[58] McAuley E, Duncan T, Tammen VV (1989). Psychometric properties of the Intrinsic Motivation Inventory in a competitive sport setting: a confirmatory factor analysis. Res Q Exerc Sport.

[59] Clancy RB, Herring MP, Campbell MJ (2017). Motivation Measures in Sport: A Critical Review and Bibliometric Analysis. Front Psychol.

[60] Barathi S, O?Neill E, Lutteroth C, Finnegan D, Farrow M, Whaley A, Heath P, Buckley J, Dowrick P, Wuensche B, Bilzon J (2018). Interactive Feedforward for Improving Performance and Maintaining Intrinsic Motivation in VR Exergaming. Proceedings of the SIGCHI Conference on Human Factors in Computing Systems.

[61] Keesing A, Ooi M, Wu O, Ye X, Shaw L, Wünsche B (2019). HIIT With Hits: Using Music and Gameplay to Induce HIIT in Exergames. Proceedings of the Australasian Computer Science Week Multiconference.

[62] Hettiarachchi IT, Hanoun S, Nahavandi D, Nahavandi S (2019). Validation of Polar OH1 optical heart rate sensor for moderate and high intensity physical activities. PLoS One.

[63] Schubert M, Clark A, De La Rosa A (2018). The Polar OH1 Optical Heart Rate Sensor is Valid during Moderate-Vigorous Exercise. Sports Med Int Open.

[64] Cohen J (1988). Statistical Power Analysis for the Behavioral Sciences.

[65] Gao Z, Podlog L, Lee J (2014). Children's situational motivation, rate of perceived exertion and physical activity levels in exergaming: Associations and gender differences. Video Games: Parents' Perceptions, Role of Social Media and Effects on Behavior.

[66] Montoye H (2000). Energy costs of exercise and sport. Nutrition in Sport.

[67] Reiff C, Marlatt K, Dengel DR (2012). Difference in caloric expenditure in sitting versus standing desks. J Phys Act Health.

[68] Malone LA, Thirumalai M, Padalabalanarayanan S, Neal WN, Bowman S, Mehta T (2019). Energy Expenditure and Enjoyment During Active Video Gaming Using an Adapted Wii Fit Balance Board in Adults with Physical Disabilities: Observational Study. JMIR Serious Games.

[69] Mat Rosly M, Mat Rosly H, Davis Oam GM, Husain R, Hasnan N (2017). Exergaming for individuals with neurological disability: a systematic review. Disabil Rehabil.

[70] Graves LE, Ridgers ND, Stratton G (2008). The contribution of upper limb and total body movement to adolescents' energy expenditure whilst playing Nintendo Wii. Eur J Appl Physiol.

[71] Lanningham-Foster L, Foster RC, McCrady SK, Jensen TB, Mitre N, Levine JA (2009). Activity-promoting video games and increased energy expenditure. J Pediatr.

[72] Lanningham-Foster L, Jensen TB, Foster RC, Redmond AB, Walker BA, Heinz D, Levine JA (2006). Energy expenditure of sedentary screen time compared with active screen time for children. Pediatrics.

[73] Mellecker RR, McManus AM (2008). Energy expenditure and cardiovascular responses to seated and active gaming in children. Arch Pediatr Adolesc Med.

[74] Wiemeyer J, Deutsch J, Malone LA, Rowland JL, Swartz MC, Xiong J, Zhang FF (2015). Recommendations for the Optimal Design of Exergame Interventions for Persons with Disabilities: Challenges, Best Practices, and Future Research. Games Health J.

[75] Bushman B (2017). ACSM's Complete Guide to Fitness & Health.

[76] Muñoz J, Cameirão M, Bermúdez i Badia S, Gouveia E (2018). Closing the Loop in Exergaming - Health Benefits of Biocybernetic Adaptation in Senior Adults. Proceedings of the Annual Symposium on Computer-Human Interaction in Play.

[77] Möhlenkamp S, Lehmann N, Breuckmann F, Bröcker-Preuss M, Nassenstein K, Halle M, Budde T, Mann K, Barkhausen J, Heusch G, Jöckel KH, Erbel R, Marathon Study Investigators, Heinz Nixdorf Recall Study Investigators (2008). Running: the risk of coronary events : Prevalence and prognostic relevance of coronary atherosclerosis in marathon runners. Eur Heart J.

