# Supplementary material for: Results and Guidelines From a Repeated-Measures Design Experiment Comparing Standing and Seated Full-Body Gesture-Based Immersive Virtual Reality Exergames: Within-Subjects Evaluation
Source: JMIR Serious Games. 2020 Jul 27;8(3):e17972. doi: 10.2196/17972 (PMC7418021; doi:10.2196/17972)
Supplement: Multimedia Appendix 1 [file games_v8i3e17972_app1.pdf]

OVERVIEW

In this section, we describe how we measure the intensity of gestures based on research from exercise science and then how we calculate the training intensity of the game.

Gesture Intensity

In exercise science, heart rate is commonly used as a metric to evaluate the intensity level of a certain exercise [1]. The corresponded intensity I_i for each exercise can be converted based on their heart rates (i.e., HR_{max}, HR_{avg}, HR_{rest}) calculated by the Karvonen formula [2].

$$HR_{avg} = (HR_{max} - HR_{rest})I_i + HR_{rest} \quad (1)$$

$$I_i = \frac{HR_{avg} - HR_{rest}}{HR_{max} - HR_{rest}} \quad (2)$$

Where $I_i \in [0,1]$; $HR_{max} = 220 - age$; HR_{avg} is the average HR measured during the exercise; HR_{rest} is the rest HR of the user.

To get the intensity I_i and corresponding heart rate HR_i, we conducted the following pilot study with a similar approach in [3]:

- First, a level consisting of an infinite number of gestures with gesture type Ges_i is provided.
- Ten healthy university students (5 females) whose age ranged from 22 to 28 were recruited to play each level for about 5 minutes. None of them were athletes.
- The experiment run over several days for each participant to mitigate any effects due to tiredness.
- We designed and tested 10 gestures for each version; the version and the gestures were counterbalanced.
- Duration D_i (in seconds) associated with gesture type Ges_i was calculated as the average time participants take to finish each gesture of that type.

Exergame Intensity Evaluation

To get the corresponding intensity level of each exergame, we used the following formula:

$$AvgI_{game} = \sum_{n=1}^{6} P_n \times I_{(n)} \times c \quad (3)$$

Where $AvgI_{game}$ is the intensity level of the corresponding game. P_n is the probability of triggering each gesture Ges_i which we have set it as $\frac{1}{6}$. $I_{(n)}$ is the intensity level when doing the gesture Ges_i, which had been measured in the pilot study. Coefficient c can be calculated by $\frac{D_i}{2.5}$ where 2.5s is the allowed action time we set for Experiment 1.

Our calculation results show that the intensities for standing and seated exergames are 31.1% and 30.1%, respectively. The six selected gestures led to a similar intensity level for both the standing and seated exergames.

REFERENCE

1. Levine BD. VO2max: what do we know, and what do we still need to know? J Physiol 2008 Jan 1;586(1):25–34. [doi: 10.1113/jphysiol.2007.147629]

2. Karvonen J, Vuorimaa T. Heart Rate and Exercise Intensity During Sports Activities. Sports Medicine 1988 May 1;5(5):303–311. [doi: 10.2165/00007256-198805050-00002]

3. Xie B, Zhang Y, Huang H, Ogawa E, You T, Yu L-F. Exercise Intensity-Driven Level Design. IEEE Trans Visual Comput Graphics 2018 Apr;24(4):1661–1670. [doi: 10.1109/TVCG.2018.2793618]
