# Supplementary material for: Results and Guidelines From a Repeated-Measures Design Experiment Comparing Standing and Seated Full-Body Gesture-Based Immersive Virtual Reality Exergames: Within-Subjects Evaluation
Source: JMIR Serious Games. 2020 Jul 27;8(3):e17972. doi: 10.2196/17972 (PMC7418021; doi:10.2196/17972)
Supplement: Multimedia Appendix 2 [file games_v8i3e17972_app2.pdf]

A: IMI Questionnaire

1	2	3	4	5	6	7
not at all			somewhat			Very

Interest/Enjoyment

1. I enjoyed doing this activity very much
2. This activity was fun to do.
3. I thought this was a boring activity. (Reverse score)
4. This activity did not hold my attention at all. (Reverse score)
5. I would describe this activity as very interesting.
6. I thought this activity was quite enjoyable.
7. While I was doing this activity, I was thinking about how much I enjoyed it.

Perceived Competence

1. I think I am pretty good at this activity.
2. I think I did pretty well at this activity, compared to other students.
3. After working at this activity for awhile, I felt pretty competent.
4. I am satisfied with my performance at this task.
5. I was pretty skilled at this activity.
6. This was an activity that I couldn't do very well. (Reverse score)

Pressure/Tension

1. I did not feel nervous at all while doing this. (Reverse score)
2. I felt very tense while doing this activity.
3. I was very relaxed in doing these. (Reverse score)
4. I was anxious while working on this task.
5. I felt pressured while doing these.

Value/Usefulness

1. I believe this activity could be of some value to me.
2. I think that doing this activity is useful for health
3. I think this is important to do because it can improve my health
4. I would be willing to do this again because it has some value to me.
5. I think doing this activity could help me to build up my body
6. I believe doing this activity could be beneficial to me.
7. I think this is an important activity.

B: Motion Sickness Assessment Questionnaire

1	2	3	4	5	6	7	8	9
Not at all								Severely

Questions:

1. I felt sick to my stomach
2. I felt faint-like
3. I felt annoyed/irritated
4. I felt sweaty
5. I felt queasy
6. I felt lightheaded
7. I felt drowsy
8. I felt clammy/cold sweat
9. I felt disoriented
10. I felt tired/fatigued
11. I felt nauseated

12. I felt hot/warm
13. I felt dizzy
14. I felt like I was spinning
15. I felt as if I may vomit
16. I felt uneasy

C: BORG 6-20 Rate of Perceived Exertion Scale (RPE)
Select one rating from the below table:

Perceived Exertion	Rating	Description
No Exertion	6	Little to no movement, very relaxed
Extremely Light	7	Able to maintain pace
	8	
Very Light	9	Comfortable and breathing harder
	10	
Light	11	Minimal sweating, can talk easily
	12	
Somewhat Hard	13	Slight breathlessness, can talk
	14	Increased sweating, still able to hold conversation but with difficulty
Hard	15	Sweating, able to push and still maintain proper form
	16	
Very Hard	17	Can keep a fast pace for a short time period
	18	
Extremely Hard	19	Difficulty breathing, near muscle exhaustion
Maximally Hard	20	STOP exercising, total exhaustion

D: Fear of falling

How concerned you are about the possibility of falling during the experiment?

1	2	3	4	5	6	7	8	9	10
Not at all									Extremely

E: Comments:

What do you think about this version of the game?
